# Hypothermia combined with neuroprotective adjuvants shortens the duration of hospitalization in infants with hypoxic ischemic encephalopathy: Meta-analysis

**DOI:** 10.3389/fphar.2022.1037131

**Published:** 2023-01-06

**Authors:** Andrea Ovcjak, Riley Pontello, Steve P. Miller, Hong-Shuo Sun, Zhong-Ping Feng

**Affiliations:** ^1^ Department of Physiology, Faculty of Medicine, University of Toronto, Toronto, ON, Canada; ^2^ Department of Pediatrics, Faculty of Medicine, University of British Columbia, Vancouver, BC, Canada; ^3^ Department of Surgery, Faculty of Medicine, University of Toronto, Toronto, ON, Canada; ^4^ Department of Pharmacology and Toxicology, Faculty of Medicine, The University of Toronto, Toronto, ON, Canada; ^5^ Leslie Dan Faculty of Pharmacy, University of Toronto, Toronto, ON, Canada

**Keywords:** hypoxic-ischemic encephalopathy, neonatal, therapeutic hypothermia, adjuvant therapy, neuroprotection, meta-analysis

## Abstract

**Objective:** Therapeutic hypothermia (TH) is the current standard of care for neonatal hypoxic-ischemic encephalopathy (HIE), yet morbidity and mortality remain significant. Adjuvant neuroprotective agents have been suggested to augment hypothermic-mediated neuroprotection. This analysis aims to identify the classes of drugs that have been used in combination with hypothermia in the treatment of neonatal HIE and determine whether combination therapy is more efficacious than TH alone.

**Methods:** A systematic search of PubMed, Embase and Medline from conception through December 2022 was conducted. Randomized- and quasi-randomized controlled trials, observational studies and retrospective studies evaluating HIE infants treated with combination therapy *versus* TH alone were selected. Primary reviewers extracted information on mortality, neurodevelopmental impairment and length of hospitalization for meta-analyses. Effect sizes were pooled using a random-effects model and measured as odds ratio (OR) or mean difference (MD) where applicable, and 95% confidence intervals (CI) were calculated. Risk of bias was assessed using the tool from the Cochrane Handbook for Systematic Reviews of Interventions.

**Results:** The search strategy collected 519 studies, 16 of which met analysis inclusion criteria. HIE infants totaled 1,288 infants from included studies, 646 infants received some form of combination therapy, while 642 received TH alone. GABA receptor agonists, NMDA receptor antagonists, neurogenic and angiogenic agents, stem cells, glucocorticoids and antioxidants were identified as candidate adjuvants to TH that have been evaluated in clinical settings compared to TH alone. Length of hospitalization was significantly reduced in infants treated with combination therapy (MD −4.81, 95% CI [−8.42. to −1.19], *p* = .009) compared to those treated with TH alone. Risk of mortality and neurodevelopmental impairment did not differ between combination therapy and TH alone groups.

**Conclusion:** Compared to the current standard of care, administration of neuroprotective adjuvants with TH reduced the duration of hospitalization but did not impact the risk of mortality or neurodevelopmental impairment in HIE infants. Meta-analysis was limited by a moderate risk of bias among included studies and small sample sizes. This analysis highlights the need for preclinical trials to conduct drug development studies in hypothermic settings to identify relevant molecular targets that may offer additive or synergistic neuroprotection to TH, and the need for larger powered clinical trials to determine the dose and timing of administration at which maximal clinical benefits are observed for adjuvant neuroprotectants.

## 1 Introduction

Although substantial progress has been made in reducing neonatal mortality rates since 1990, annual neonatal deaths remain remarkably high with socioeconomic and geographic disparities continuing to widen. While improvements in health and maternal care have led to rapid reductions in deaths caused by infection (i.e., meningitis, sepsis, tetanus), perinatal asphyxia–the main reason for hypoxic-ischemic encephalopathy (HIE)—has continued to account for nearly a quarter of neonatal mortality for the past 3 decades (Lawn et al., 2005; Lawn et al., 2010; Hug et al., 2019). In high-income regions, HIE has an estimated incidence of 1.5/1,000 live births, with death or long-term neurological sequalae reported in upwards of 60% of infants (Edwards et al., 2010; Kurinczuk et al., 2010; Lundgren et al., 2018). This suggests that in Canada, ∼540 infants were affected by this condition in the year 2020 alone (Statistics Canada, 2022). HIE is thus an important contributor to the burden of death and disability in the population, with considerable socioeconomic implications.

The underlying etiology of HIE is characterized by a drop in cerebral blood flow and/or hypoxemia with the resulting pathophysiology progressing in distinct phases (Millar et al., 2017). Primary depletion of high energy phosphates drives anoxic depolarization and early cell death, followed by a transient recovery period due to blood reperfusion. Approximately 6–15 h later, there is a secondary surge of delayed cell death that is driven by excitotoxicity, mitochondrial failure and oxidative stress, and is accompanied by chronic inflammation, seizures and cytotoxic edema (Fatemi et al., 2009; Cotten and Sankaran, 2010; Yıldız et al., 2017). These deleterious effects are compounded by the intrinsic vulnerability of the neonatal brain attributed, in part, to immature antioxidant defense mechanisms and rising metabolic demands as the cerebral energy source shifts from anaerobic glycolysis to aerobic metabolism in order to fuel complex maturational processes (Liu et al., 2014; Martini et al., 2021). Clinical magnetic resonance spectroscopy studies have supported this biphasic model of cell death within the brain. In term and near-term infants with evidence of birth asphyxia and moderate to severe HIE, normal cerebral metabolism is observed shortly after birth, followed by a period of secondary energy failure, the severity of which is correlated with histological manifestation of brain damage and neurodevelopmental outcome at 4 years of age (Wyatt et al., 1989; Roth et al., 1997). Thus targeting secondary cell death mechanisms is opportune for therapeutic intervention.

Therapeutic hypothermia (TH) was the first empirically supported neuroprotective treatment for neonates with HIE and has become the clinical standard of care. Hypothermic therapy was derived from its ability to reduce brain metabolism by ∼5% per 1°C below normothermic levels, subsequently suppressing various delayed cell death mechanisms (Laptook et al., 1995). Accordingly, in clinical settings, selective head or whole-body cooling to 33°C–34°C, instituted within 6 h of birth and continued for up to 72 h, reduces the combined risk of death or neurodevelopmental disability at 18 months of age by ∼11% (Edwards et al., 2010). However meta-analysis of eight clinical trials determined that TH has a number needed to treat of seven for this composite outcome, and a more recent randomized controlled trial showed that TH for HIE infants did not have a statistically significant effect on mortality at 6–7 years of age (Jacobs et al., 2013; Azzopardi et al., 2014). Hence, a current focus of research has been the exploration of adjunct therapies to TH, that target the same or different pathophysiological mechanisms of secondary injury, thereby exerting synergistic or additive neuroprotective effects, respectively.

Various pharmacologic agents have been associated with neuroprotection in animal models of HIE and accumulating evidence at both experimental and clinical levels have demonstrated the potential for select drugs to augment hypothermic-mediated neuroprotection (Cho et al., 2020; Zhou et al., 2020). The relative efficaciousness of hypothermia combined adjuvant drugs compared to TH alone in clinical settings however, remains largely unknown (Razak and Hussain, 2019; Ahmed et al., 2021). Thus, there exists a need for a quantitative comparison of the efficacy of TH alone vs. combination therapy, in which the latter consists of the most promising candidate adjuvants to TH. This will allow us to identify the clinical utility of combination therapy for the treatment of HIE in the current era, as well as identify whether certain classes of drugs–targeting certain pathophysiological mechanisms–confer greater outcomes in clinical settings. The objective of the present analysis is to, 1) characterize the classes of drugs that have been used in combination with TH in the treatment of neonatal HIE; 2) determine the effect of combination therapy and TH on mortality, long-term neurodevelopmental impairment and length of hospitalization–outcome measures that predict personal and healthcare burdens; 3) identify whether certain classes of drugs represent a more effective adjunctive therapy to enhance the neuroprotective effects of TH in treating neonatal HIE.

## 2 Materials and methods

### 2.1 Publication selection

#### 2.1.1 Types of studies

Randomized- and quasi-randomized controlled trials, observational studies and retrospective studies were considered eligible for inclusion.

#### 2.1.2 Types of participants

Eligible studies must have reported data collected from human infants who met the following criteria: 1) ≥35 weeks gestational age (term, near-term infants); 2) evidence of moderate or severe HIE; 3) met the physiologic eligibility criteria for TH, with treatment initiated within 6 h of life. Diagnostic criteria for HIE varied between hospitals but generally included an Apgar score <5 during the first 10 min of life and/or assisted ventilation, as well as moderate/severe encephalopathy as evidenced by modified Sarnat criteria, abnormal neurological signs and/or abnormal amplitude-integrated electroencephalogram (aEEG). Studies excluded infants with major congenital and hereditary abnormalities, congenital viral infections, or evidence of overt encephalopathy other than HIE.

#### 2.1.3 Types of interventions

Eligible studies must have included two intervention groups: combination therapy (defined as, therapeutic agent and TH) vs*.* TH alone. The therapeutic agent must have been administered for the first time within the first 24 h of life. The method of TH must have been consistent between the intervention groups and initiated within 6 h of life. Supportive therapy (control of seizures, maintenance of normal ventilation, blood glucose) was administered to infants at the discretion of attending physicians when required.

#### 2.1.4 Types of outcomes

Eligible studies must have reported at least one of the following outcome measures:

1) Mortality: assessed as death during the neonatal–infancy period.
2) Neurodevelopmental impairment (NDI): assessed by any form of standardized, validated tool or scoring system during the neonatal–infancy period.
3) Length of hospitalization: assessed as duration of time spent in hospital before discharge, measured in days.

### 2.2 Review methods

#### 2.2.1 Search strategy

Searches were conducted using the electronic databases PubMed, Embase and Medline. Preliminary literature review had led to the identification of various therapeutic agents shown to exert neuroprotection in experimental and clinical studies of neonatal HIE. The identified therapeutic agents led to the search terms used for all the databases which were as follows: hypothermia AND Erythropoietin AND neonatal hypoxic ischem*; hypothermia AND stem cells AND neonatal hypoxic ischem*; hypothermia AND Phenobarbital AND neonatal hypoxic ischem*; hypothermia AND Levetiracetam AND neonatal hypoxic ischem*; hypothermia AND Dizocilpine AND neonatal hypoxic ischem*; hypothermia AND xenon AND neonatal hypoxic ischem*; hypothermia AND topiramate AND neonatal hypoxic ischem*; hypothermia AND N-Acetylcysteine AND neonatal hypoxic ischem*; hypothermia AND allopurinol AND neonatal hypoxic ischem*; hypothermia AND crocin AND neonatal hypoxic ischem*; hypothermia AND cannabinoid AND neonatal hypoxic ischem*; hypothermia AND melatonin AND neonatal hypoxic ischem*; hypothermia AND connexinhemichannel blockade AND neonatal hypoxic ischem*; hypothermia AND magnesium AND neonatal hypoxic ischem*; hypothermia AND IGF-1 AND neonatal hypoxic ischem*; hypothermia AND hydrocortisone AND neonatal hypoxic ischem*; hypothermia AND Dexmedetomidine AND neonatal hypoxic ischem*; hypothermia AND caffeine AND neonatal hypoxic ischem*; hypothermia AND Darbepoetin AND neonatal hypoxic ischem*. References of eligible studies were searched to identify additional relevant studies that did not appear based on the search terms. All searches were inclusive of studies published from inception up to December 2022.

#### 2.2.2 Data extraction

Following the initial publication search, all titles and abstracts were screened, and a final list of studies selected for full-text review was assimilated. To assess eligibility for inclusion, two investigators independently read and extracted data from each study using a pre-determined template. Extracted data included study design, sample size, sample sex, therapeutic agent characteristics, TH protocol, and outcome measures. Once eligible studies were identified, the two investigators determined the primary mechanism of action for each utilized therapeutic agent, and studies were accordingly categorized by class of drug.

#### 2.2.3 Assessment of risk of bias

The methodological quality of the included studies was evaluated independently by two investigators using the risk of bias tool from the Cochrane Handbook for Systematic Reviews of Interventions (Higgins et al., 2011). Assessments were made in the following domains: random sequence generation, allocation concealment, blinding of participants, personnel and outcome assessors, incomplete outcome data, selective outcome reporting, and other sources of bias. All included studies passed the qualitative analysis.

#### 2.2.4 Data analysis

Using Cochrane statistical package, RevMan5.3 software, a random effect model meta-analysis was performed for each outcome measure to define differences between combination therapy- and TH alone-treated HIE infants. For outcome measures, 1) mortality and 2) NDI, a random effect model under Mantel-Haenszel methods was used to pool data across studies to calculate an estimate effect size. The comparative effect sizes were calculated as odds ratios (OR). For outcome measure, 3) length of hospitalization, studies reported continuous data and thus, within-group means and corresponding S.D. were utilized. A random effect model under inverse variance methods was used to pool data across studies to calculate an estimate effect size. The comparative effect size was calculated as mean difference (MD) as all studies reported the outcome measure using the same scale.

The 95% confidence interval (CI) was calculated for each effect size, and a two-tailed *p* < .05 was considered statistically significant (Deeks and Higgins, 2007). Between studies heterogeneity was assessed using *I* ^2^ and Cochrane’s Q method.

Publication bias was assessed using a funnel plot, with SE (log [OR]) plotted against the OR of the included studies or SE (MD) plotted against the MD of the included studies.

## 3 Results

### 3.1 Publication selection

**FIGURE 1:**
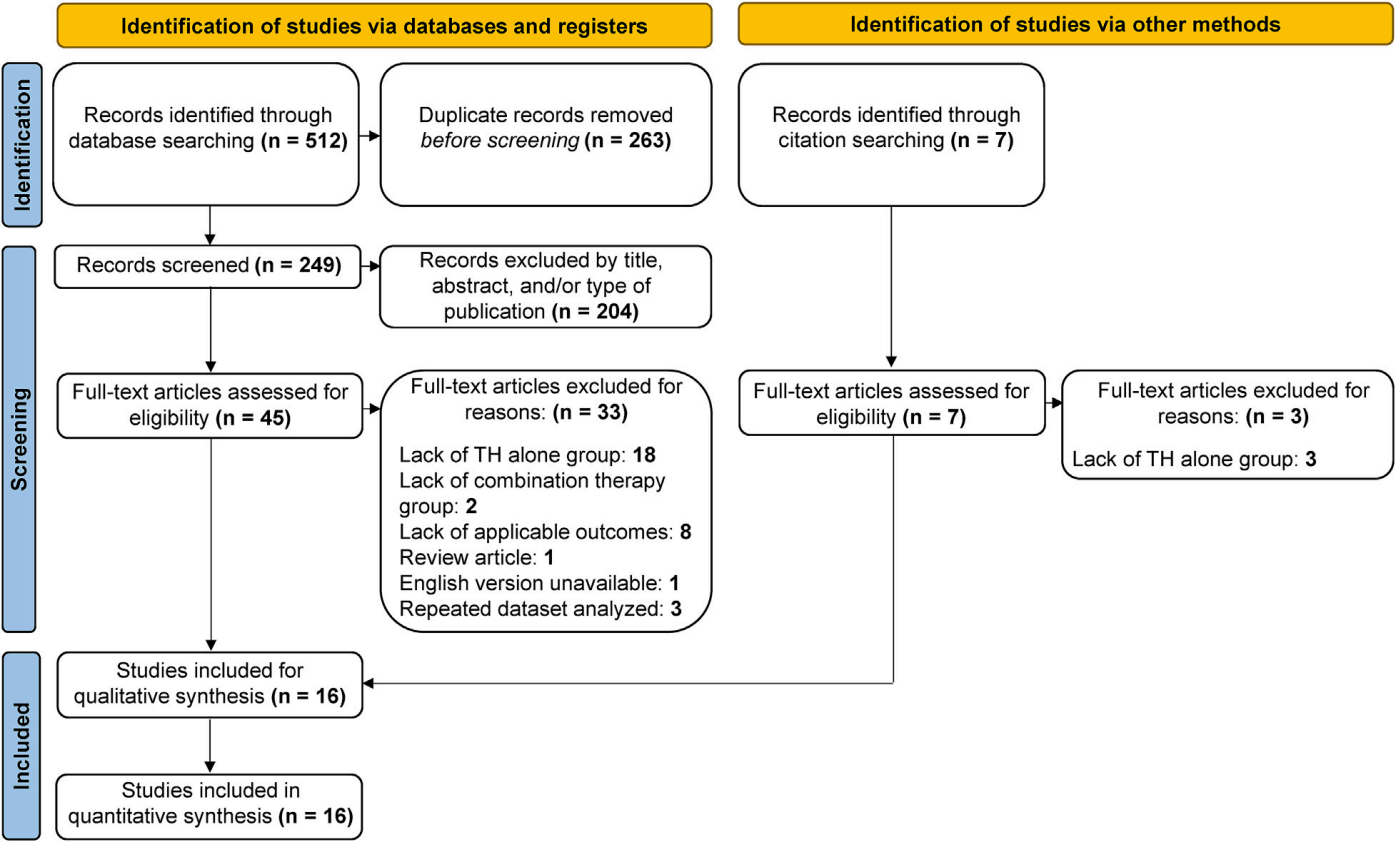
Flow diagram of study screening process. The search strategy resulted in 519 articles, 263 of which were duplicates, leaving 256 for screening. Of these, 204 were deemed irrelevant, leaving 52 articles for full-text review. A total of 16 studies met the predetermined inclusion criteria and were included in the present analysis.

A total of 519 studies were collected using electronic databases and citation searching, 263 of which were duplicates, leaving 256 studies for further screening (Figure 1). A further 204 studies were excluded by title, abstract, and/or type of publication, leaving 52 studies for full-text review. Of these, 36 studies were excluded after a full text review: 21 for a lack of a TH alone control group, two due to a lack of a combination therapy group, eight due to a lack of applicable outcome measures, one review article, one article not available in the English language, and three due to a repeated dataset analyzed. In all, 16 studies were included for meta-analysis. Study characteristics are described in Table 1. It is important to note that two studies analyzed the same dataset with different outcome measures reported (Wu et al., 2016; Mulkey et al., 2017) Accordingly, at no point are both studies included in the same meta-analysis.

**TABLE 1:** Study characteristics.

Ref	TH protocol			Experimental groups						Outcome measures		
				Treatment					Control			
	Method	Depth	Duration	Combination	Dose	Timing of 1st dose	Number of doses	Route		Mortality	NDI	Length of hospitalization
GABA Receptor Agonists												
Meyn et al. (2010)	WBC (Blanketrol Hyper-Hypothermia cooling system)	Body temperature of 33.5°C	72 h within 6 h of birth	Phenobarbital + TH	40 mg/kg	<6 h of birth, TH initiation	1	N/A	TH	Death at 18–49 months	N/A	Days until discharge
Sarkar et al. (2011)	SHC (cool cap) or WBC (Blanketrol Hyper-Hypothermia cooling system)	SHC: rectal temperature of 34.0°C–35.0°C; WBC: esophageal temperature of 33.5°C	72 h within 6 h of birth	Phenobarbital + TH	24.4 ± 6.3 mg/kg	<6 h of birth, before initiation of TH	21/34: 1 Dose; 13/34: 2 Doses (during TH)	N/A	TH	Death during neonatal period	N/A	N/A
Filippi et al. (2010)	Deep WBC or Mild WBC (ice packs; cooling blanket; Blanketrol Hyper-Hypothermia cooling system)	Deep WBC: rectal temperature of 30.0°C–33.0°C Mild WBC: rectal temperature of 32.0°C–34.0°C	72 h within 6 h of birth	Topiramate + TH	5 mg/kg/dose or 5 mg/kg on 1st day and 3 mg/kg on following 2 days	<6 h of birth, at TH initiation	3 (DOL 1–3)	Orogastric tube	TH	Death during hospitalization	N/A	N/A
Filippi et al. (2018)	WBC (Blanketrol Hyper-Hypothermia cooling system)	Esophageal temperature of 33.5°C	72 h within 6 h of birth	Topiramate + TH	10 mg/kg/dose	<6 h of birth, at TH initiation	3 (DOL 1–3)	Orogastric tube	TH	Death at 24 months	18–24 months; Severe NDD if 1 or more: developmental quotient <70, moderate or severe CP, cortical visual impairment defined as bilateral blindness caused by damage to CNS, or bilateral sensorineural hearing loss defined >40 dB reduction	N/A
Nuñez-Ramiro et al. (2019)	WBC (servo-controlled hypothermia mattresses)	Rectal temperature of 33.5°C ± .5°C	72 h	Topiramate + TH	5 mg/kg on 1st day and 3 mg/kg/day on following 5 days	at TH initiation	6 (DOL 1–6)	Nasogastric tube	TH + Placebo (sterile water)	Death before discharge	N/A	N/A
NMDA Receptor Antagonists												
Rahman et al. (2015)	SHC (cool cap) or WBC	Rectal temperature of 33.0°C–34.0°C	72 h within 6 h of birth	MgSO4+TH	250 mg/kg/dose	<6 h of birth	3 (DOL 1–3)	Intravenous over 30 min	TH + Placebo (.9% saline)	Death at discharge from hospital	N/A	N/A
Gulczynska et al. (2018)	SHC (cool cap) or WBC (Inspiration Healthcare/Tecotherm Neo)	SHC: rectal temperature of 34.0°C–35.0°C; WBC: rectal temperature of 33.0°C–34.0°C	72 h within 6 h of birth	MgSO4+TH	250 mg/kg/dose	<6 h of birth	3 (DOL 1–3)	Infusion over 60 min	TH	Death during hospital stay	N/A	Age at discharge
Azzopardi et al. (2019)	Cooling (servo-controlled equipment)	Rectal temperature of 33·5°C	72 h within 6 h of birth	Xenon + TH	30% xenon mixture	<12 h of birth	Continuous for 24 h	Endotracheal tube	TH	Death at 2–3 years	2–3 years; Moderate disability: BSID-III 56–69, GMFCS 2/3, or moderately reduced vision; Severe disability: BSID-III 55, GMFCS 4/5, or no useful vision	N/A
Neurogenic and Angiogenic Agents												
Baserga et al. (2015)	SHC or WBC	N/A	72 h within 6 h of birth	Darbepoetin alfa + TH	2 ug/kg/dose or 10 ug/kg/dose	<12 h of birth	2 (DOL 1, 7)	Intravenous over 5 min	TH + Placebo (normal saline)	Death in first month of life	N/A	N/A
Wu et al. (2016)	SHC or WBC	N/A	within 6 h of birth	Erythropoietin + TH	1000 U/kg/dose	<24 h of birth	5 (DOL 1, 2, 3, 5, 7)	Intravenous	TH + Placebo (normal saline)	Death before hospital discharge	12 months; Moderate-severe NDI: AIMS score less than 5th percentile for age or WIDEA score <76.4	N/A
Mulkey et al. (2017)	SHC or WBC	N/A	within 6 h of birth	Erythropoietin + TH	1000 U/kg/dose	<24 h of birth	5 (DOL 1, 2, 3, 5, 7)	Intravenous	TH + Placebo (normal saline)	N/A	N/A	Length of hospital stay
Lv et al. (2017)	SHC (HGT-2000 therapeutic instrument)	Nasopharyngeal temperature of 33.5°C–34.0°C	72 h	Recombinant human Erythropoietin + TH	200 IU/kg/dose in 10% glucose solution	On second day of hospitalization	10 (DOL 2–11)	Intravenous	TH	N/A	9 months; Gross motor neurodevelopment retardation: GDS developmental quotient <75	N/A
Wu et al. (2022)	WBC	N/A	72 h within 6 h of birth	Erythropoietin + TH	1000 U/kg/dose	<26 h of birth	5 (DOL 1, 2, 3, 4, 7)	Intravenous	TH + Placebo (normal saline)	Death at 22–36 months	22–36 months: CP, GMFCS ≥1 or BSID-III <90	
Stem Cells												
Cotton et al. (2014)	WBC	33.5°C	72 h	Nucleated umbilical cord blood (UCB) cells + TH	1–5 × 107 cells/kg/dose	As soon as possible after birth	Up to 4 (DOL 0–3)	Infusion	TH	Death at 15 months	12 months; Moderate-severe NDD: BSID-III <85, or could not be scored due to severe impairment	N/A
Glucocorticoids												
Kovacs et al. (2019)	WBC	Rectal temperature of 33.0°C–34.0°C	72 h within 6 h of birth	Hydrocortisone + dopamine + TH	.5 mg/kg/dose	<12 h of birth	13 (Every 6 h after TH initiation)	Intravenous	TH + Placebo + dopamine	In-hospital death	N/A	N/A
Antioxidants												
Aly et al. (2014)	WBC (ambient temperature exposure and ice packs)	Rectal temperature of 33.0°C–34.0°C	72 h within 6 h of birth	Melatonin + TH	10 mg/kg/dose	<24 h of birth	5 (DOL 1–5)	Orogastric tube<24 h	TH	Death at 6 months	6 months; Abnormal DDST-II based on gross motor, language, fine motor-adaptive and personal-social test items	N/A

N/A indicates data was not available. Abbr.: TH, therapeutic hypothermia; SHC, selective head cooling; WBC, whole-body cooling; DOL, days of life, NDD: neurodevelopmental disability; CP, cerebral palsy; BSID-III, bayley scales of infant and toddler development, third edition; GMFCS, gross motor function classification system; AIMS, alberta infant motor scale; WIDEA, warner initial developmental evaluation; DDST-II, Denver Developmental Screening Test II.

### 3.2 Study subgroups based on drug class

In addition to identifying whether combination therapy improves outcome measures to a greater extent than TH alone, we sought to investigate if there were certain classes of therapeutic agents that served as more efficacious adjuvants to TH than others. Thus, the therapeutic agents combined with TH were categorized based on their mechanism of action, and studies were accordingly sorted into subgroups (Table 2). Doing so allowed for preliminary conclusions to be drawn regarding the pathophysiological mechanisms during HIE progression in the brain that are most important to target in the setting of hypothermic temperatures. See the identified subgroups described below.

**TABLE 2:** Study subgroups. Classification based on drug class of the therapeutic agent in the combination therapy group.

Study	Drug in combination therapy	Drug class	Mechanism of action
Meyn et al. (2010)	Phenobarbital	GABA Receptor Agonists	Activates or potentiates GABAR-mediated Cl^−^ currents, thereby reducing neuronal excitability and metabolic by-product overloading of cells
Sarkar et al. (2011)	Phenobarbital		
Filippi et al. (2010)	Topiramate		
Filippi et al. (2018)	Topiramate		
Nuñez-Ramiro et al. (2019)	Topiramate		
Rahman et al. (2015)	MgSO4	NMDA Receptor Antagonists	Inhibits NMDA receptors, reducing extracellular Ca^2+^ influx and accumulation of toxic metabolites
Gulczynska et al. (2018)	MgSO4		
Azzopardi et al. (2019)	Xenon Gas		
Baserga et al. (2015)	Darbepoetin alpha	Neurogenic and Angiogenic Agents	Stimulates neurogenesis and angiogenesis to promote remodeling and recovery of cell functioning
Wu et al. (2016)	Erythropoietin		
Mulkey et al. (2017)	Erythropoietin		
Lv et al. (2017)	Erythropoietin		
Wu et al. (2022)	Erythropoietin		
Cotten et al. (2014)	UBC cells	Stem Cells	Upregulates growth factors and promotes neural and vascular repair
Kovacs et al. (2019)	Hydrocortisone	Glucocorticoids	Inhibits inflammatory pathways, activates anti-inflammatory mediators and generates systemic immunosuppression
Aly et al. (2014)	Melatonin	Antioxidants	Reduces oxidative stress within the cell by scavenging destructive free radicals and promoting antioxidant enzyme expression

#### 3.2.1 GABA receptor agonists

Phenobarbital (PB) (Meyn et al., 2010; Sarkar et al., 2011) or topiramate (TPM) (Filippi et al., 2010; 2018; Nuñez-Ramiro et al., 2019) were administered concurrently with TH in the combination therapy groups of five studies. PB and TPM are anticonvulsants with particular effectiveness against focal and generalized tonic-clonic seizures (Abou-Khalil, 2019). Both drugs have been used independently to treat HIE-related seizures in clinical settings. PB is a long-acting barbiturate that binds an allosteric site on the γ-aminobutyric acid (GABA)-A receptor, activating and prolonging the duration of opening of the associated chloride channel (Pacifici, 2016). TPM is a sulfate-substituted monosaccharide that works to potentiate GABA-induced Cl^−^ currents (Shank et al., 2000). PB and TPM thus work to induce membrane hyperpolarization and reduce neuronal excitability, suggesting inhibition against HIE-induced excitotoxicity.

#### 3.2.2 NMDA receptor antagonists

Magnesium sulfate (MgSO4) (Rahman et al., 2015; Gulczynska et al., 2018) or Xenon gas (Azzopardi et al., 2019) were combined with TH in three studies. MgSO4 is a clinically feasible and safe molecule recommended by the World Health Organization for antenatal administration in women at risk of preterm birth and shown to exert fetal neuroprotection (Chollat et al., 2018). MgSO4 gates N-methyl-d-aspartate (NMDA) receptors in a voltage dependent manner and protects against glutamate-mediated neurotoxicity (Nowak et al., 1984; Gathwala et al., 2010). Xenon is a noble gas approved for inhaled anesthesia and has been shown to be hemodynamically safe in human infants. Xenon exerts potent non-competitive inhibition of NMDA receptors and has been associated with the regulation of key apoptotic mediators and induction of hypoxia-inducible factor 1α (HIF-1α) (Ma et al., 2005; Sanders et al., 2005; Daqing et al., 2009). In the context of HIE, as NMDA receptor antagonists, MgSO4 and Xenon act to block excessive calcium influx, thereby maintaining calcium homeostasis within the cell and suppressing excitotoxic processes.

#### 3.2.3 Neurogenic and angiogenic agents

Erythropoietin (Epo) (Wu et al., 2016; Lv et al., 2017; Mulkey et al., 2017; Wu et al., 2022) or darbepoetin alpha (Dpo) (Baserga et al., 2015) were utilized alongside of TH in the combination therapy group of five studies. Epo is a glycoprotein produced by peritubular fibroblasts in the kidneys and acts on the receptor, EpoR, expressed throughout the brain in neurons, glial cells and endothelial cells (Rangarajan and Juul, 2014). While Epo is an endogenous growth factor that promotes the maturation of erythroid progenitors into red blood cells, Dpo is a synthetic hyperglycosylated Epo analog. Although Dpo has a longer half-life and decreased clearance compared to Epo, both are expected to have similar downstream effects (Sinha et al., 2019). In rodent stroke models, Epo treatment has been shown to preserve brain structure and promote neurogenesis and oligodendrogenesis at the lesion site by increasing progenitor proliferation, stimulating growth factors such as brain-derived neurotrophic factor (BDNF), and decreasing precursor cell death (Chen et al., 2007; Gonzalez et al., 2013). Epo has also been shown to upregulate vascular endothelial growth factor (VEGF), preserve blood-brain barrier integrity and increase vascular density in the brain following injury (Wang et al., 2008; Rangarajan and Juul, 2014). Thus, Epo and Dpo treatments are expected to enhance neurogenesis and angiogenesis post-HIE, promoting brain remodeling and recovery after the secondary phase of injury progression.

#### 3.2.4 Stem cells

Non-cryopreserved autologous volume- and red blood cell-reduced umbilical cord blood (UCB) cells (Cotten et al., 2014) were administered with TH in one study. UCB cells are adult stem cells derived from the human umbilical cord, and have been shown *in vitro* to express various marker proteins for early neural precursors, as well as neurons, astrocytes and oligodendrocytes (Sanchez-Ramos et al., 2001; Rosenkranz and Meier, 2011; Ballen et al., 2013). UCB cells also secrete chemokines, cytokines and growth factors including BDNF and VEGF. *In vivo*, transplantation post-HI brain injury improved sensorimotor recovery in neonatal rodents and reduced neuronal cell death (Pimentel-Coelho et al., 2010; Rosenkranz and Meier, 2011). Administration of UCB cells is thereby hypothesized to induce a regenerative environment, facilitating both neural and vascular plasticity and repair.

#### 3.2.5 Glucocorticoids

Infants were treated with hydrocortisone (Kovacs et al., 2019) combined with TH in one study. Therapeutic hydrocortisone is a synthetic analog of the endogenous hormone secreted by the adrenal cortex. Hydrocortisone binds to the glucocorticoid receptor to induce downstream effects including vasodilation, inhibition of the NF-κB inflammatory pathway and activation of anti-inflammatory mediators such as interleukin-10 (Yasir et al., 2021). Hydrocortisone is frequently utilized to treat vasopressor-resistant hypotension in preterm infants and has more recently been suggested as a neuroprotectant in models of brain injury (Higgins et al., 2009; Roquilly et al., 2013). Hydrocortisone treatment is expected to target the robust neuroinflammatory response characteristic of HIE.

#### 3.2.6 Antioxidants

Melatonin (*N*-acetyl-5-methoxytryptamine) (Aly et al., 2014) was utilized as an adjuvant to TH in the combination therapy group of one study. Melatonin is an indolamine primarily synthesized in the pineal glands that can act either by interacting with melatonin receptors, MT1 and MT2, expressed throughout the brain, or as a direct effector molecule (Dubocovich and Markowska, 2005). Melatonin is a free radical scavenger, chelating reactive oxygen and nitrogen species, while exerting potent antioxidant effects including upregulating superoxide dismutase and glutathione peroxidase (Dubocovich and Markowska, 2005; Lee et al., 2019). Following HI insult to the brain, reduced energy metabolism and cytotoxicity lead to the production of reactive oxygen species and oxidative stress within the cell. Melatonin is expected to protect against injury by promoting the expression of antioxidant enzymes and scavenging destructive free radicals.

### 3.3 Study participants

As mentioned, Wu et al. (Wu et al., 2016), and Mulkey et al. (Mulkey et al., 2017), analyzed the same dataset with different outcome measures reported. Thus to avoid duplication, only study participant data from the original trial reported by Wu et al. was included (Wu et al., 2016). The data used for the present analysis was derived from a total of 1,288 patients (695 males, 533 females, 60 unknown sex) with an average age of 38.8 weeks gestation. Infants were diagnosed with moderate (*n* = 697), severe (*n* = 363) or unspecified severity (228 patients) HIE. Modified Sarnat scoring was used to evaluate the severity of HIE in seven studies for 862 infants (Aly et al., 2014; Rahman et al., 2015; Wu et al., 2016; Lv et al., 2017; Gulczynska et al., 2018; Nuñez-Ramiro et al., 2019; Wu et al., 2022). Abnormal neurological signs and/or aEEG was used to evaluate the severity of HIE in four studies for 198 infants (Baserga et al., 2015; Filippi et al., 2018; Azzopardi et al., 2019; Kovacs et al., 2019). Finally, HIE severity scoring was not reported in four studies for 228 infants (Filippi et al., 2010; Meyn et al., 2010; Sarkar et al., 2011; Cotten et al., 2014).

A total of 642 infants received some form of combination therapy, while 646 infants received TH alone. TH protocols were similar across studies, with cooling initiated <6 h after birth, continued for 72 h, and reaching target temperatures ranging from 30°C to 35.0°C. Some studies did not report timing of initiation of TH (Aly et al., 2014; Cotten et al., 2014; Lv et al., 2017; Gulczynska et al., 2018; Nuñez-Ramiro et al., 2019), and four studies did not report the depth of hypothermia reached (Baserga et al., 2015; Wu et al., 2016; Mulkey et al., 2017; Wu et al., 2022). Regarding method of TH induction, all infants received either selective head or whole-body cooling; 138 received selective head cooling, 1,058 received whole body cooling, and one study for a total of 92 infants did not report which of the two methods infants received (Azzopardi et al., 2019). When categorized by drug class, 158 infants received GABA receptor agonists (PB, TPM), 113 received NMDA receptor antagonists (xenon gas, MgSO4), 322 received a neurogenic and angiogenic agent (Epo, Dpo), 18 received stem cells (UCB cells), 16 received a glucocorticoid (hydrocortisone) and 15 received an antioxidant (melatonin). Mortality was compared between 621 combination therapy-treated infants and 626 TH alone-treated infants. NDI was compared between 330 combination therapy-treated infants and 344 TH alone-treated infants. Length of hospitalization was compared between 78 combination therapy-treated infants and 83 TH alone-treated infants.

### 3.4 Mortality

**FIGURE 2:**
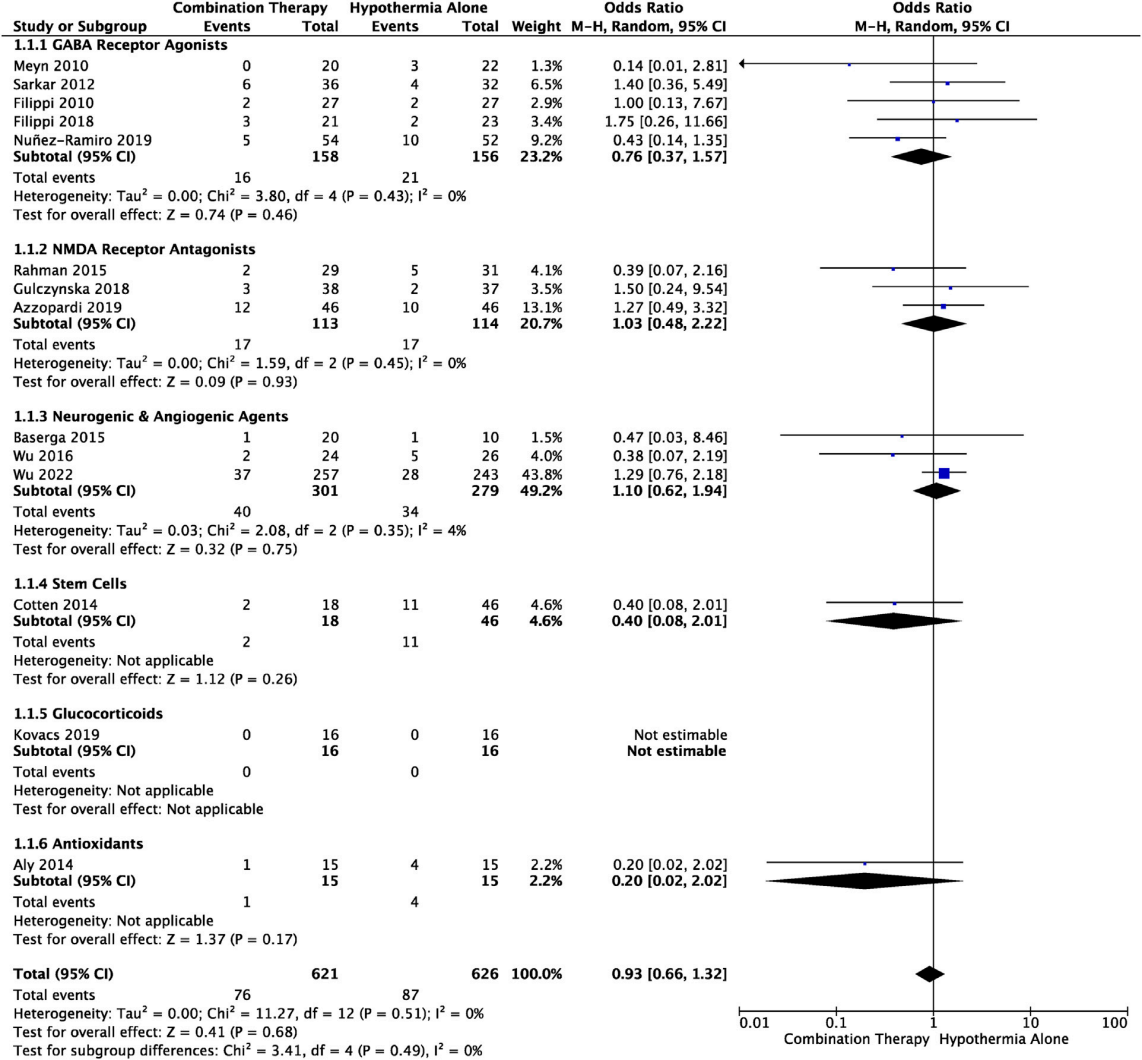
Forest plot of mortality in HIE infants treated with combination therapy compared to TH alone. An OR < 1 suggests combination therapy is more favourable to reduce the risk of mortality. Timepoint at which mortality was assessed is outlined in Table 1. OR .93, 95% CI [.66 to 1.32], *p* = .68.

Death during the neonatal to infancy period was reported in 14 studies (Table 1). The timepoint at which death was reported varied: six studies reported incidence of death before discharge from hospital, two studies reported death during the first 4 weeks of life, and six studies reported death at long-term follow-up which ranged between 6–49 months of age. Meta-analysis of all trials showed that mortality in HIE infants receiving combination therapy vs*.* TH alone, did not significantly differ (OR .93, 95% CI [.66 to 1.32], *p* = .68) (Figure 2). Subgroup analyses based on drug class revealed decreased likelihood of death in the combination therapy groups that utilized GABA receptor agonists (OR .76, 95% CI [.37 to 1.15]), stem cells (OR .40, 95% CI [.08, 2.01]) and antioxidants (OR .20, 95% CI [.02 to 2.02]), compared to TH alone groups. Conversely, there was an increased likelihood of death in NMDA receptor antagonist (OR 1.03, 95% CI [.48 to 2.22]), and neurogenic and angiogenic agent (OR 1.10, 95% CI [.13 to 1.20]) combination therapy groups compared to TH alone groups. The observed differences were not statistically significant. Among all trials, there was no statistically significant heterogeneity (I^2^ = 0).

### 3.5 Neurodevelopmental impairment (NDI)

**FIGURE 3:**
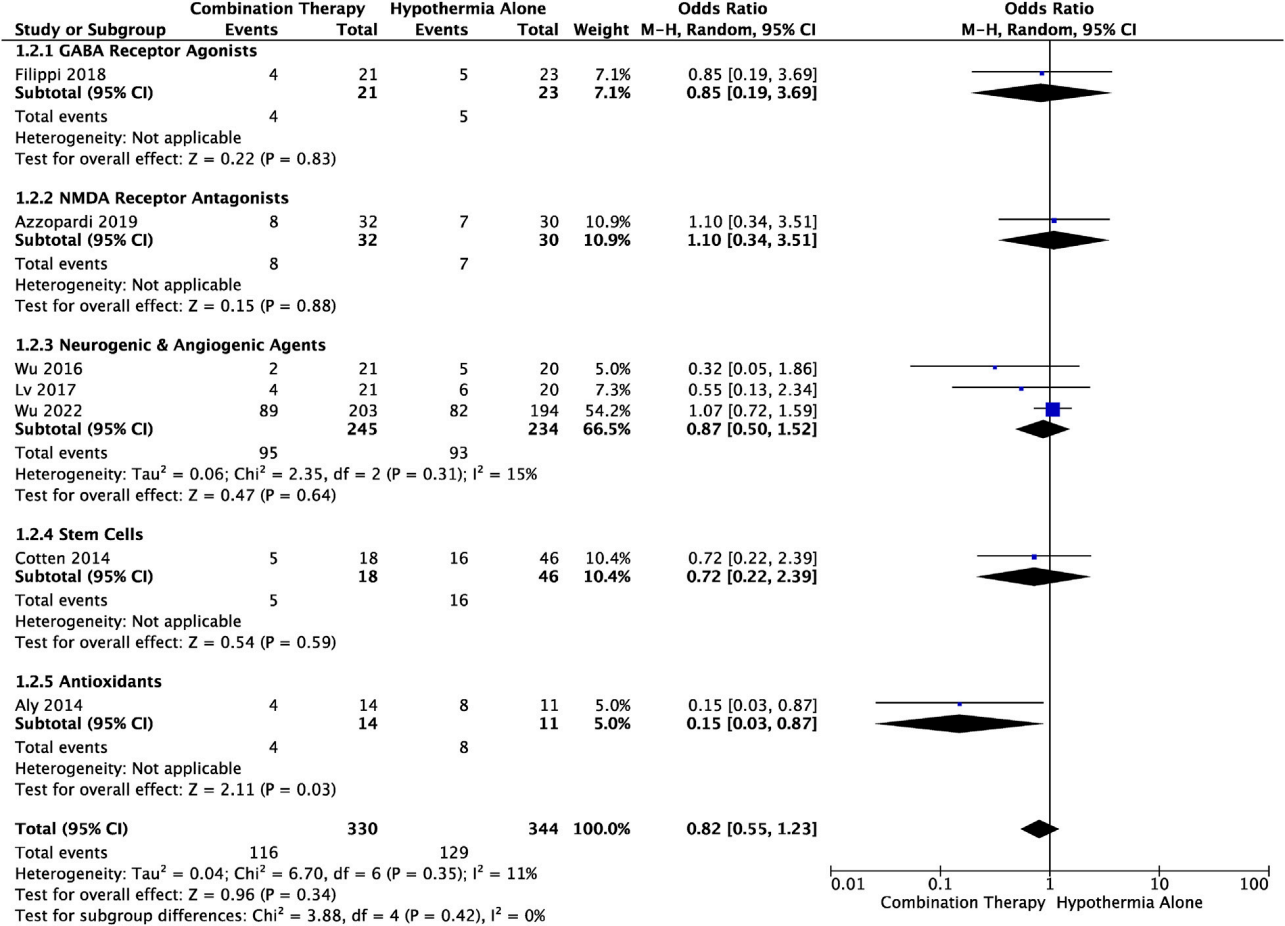
Forest plot of NDI in HIE infants treated with combination therapy compared to TH alone. An OR < 1 suggests combination therapy is more favourable to reduce the risk of an NDI diagnosis. Standardized tests used to diagnose NDI and timepoint of diagnosis for each study are outlined in Table 1. OR .82, 95% CI [.55 to 1.23], *p* = .34. Combination therapy with an antioxidant significantly reduced risk of NDI: OR .15, 95% CI [.03 to .87], *p* = .03.

There were seven studies that reported NDI during the neonatal–infancy period. Criteria for NDI, and follow-up timepoint varied between the trials and is outlined in Table 1. Analysis revealed that a smaller proportion of infants treated with combination therapy received a diagnosis of NDI compared to those treated with TH alone, although the difference was not significant (OR .61, 95% CI [.34 to 1.08], *p* = .09) (Figure 3). This trend was observed in GABA receptor agonists (OR .85, 95% CI [.19 to 3.69]), neurogenic and angiogenic agents (OR .87, 95% CI [.50 to 1.52]) and stem cells (OR .72, 95% CI [.22, 2.39]) subgroups, and was significant in the antioxidants (OR .15, 95% CI [.03 to .87], *p* = .03) subgroup. A greater portion of infants however suffered from NDI when treated with combination therapy utilizing an NMDAR antagonist compared to TH alone, although the difference was not significant (OR 1.10, 95% CI [.34 to 3.51]). Heterogeneity among all studies was not significantly different (I^2^ = 0).

### 3.6 Length of hospitalization

**FIGURE 4:**
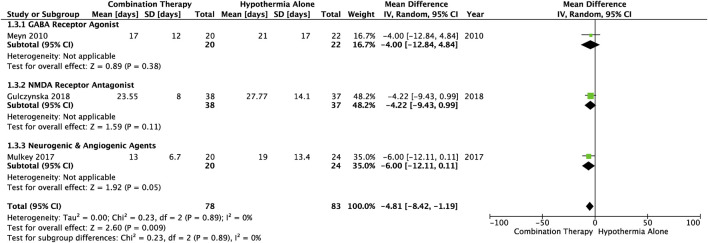
Forest plot of length of hospitalization in HIE infants treated with combination therapy compared to TH alone. Duration reported as mean (standard deviation). Combination therapy-treated infants spent significant less days in the hospital post-birth compared to infants receiving hypothermia alone: MD −4.81, 95% CI [−8.42. to −1.19], *p* = .009.

Average number of days spent in hospital from birth to discharge, was reported in three studies (Table 1). The length of hospitalization was significantly decreased in HIE infants treated with combination therapy vs*.* TH alone (MD −4.81, 95% CI [−8.42. to −1.19], *p* = .009) (Figure 4). This outcome measure included one study from each of the following subgroups: GABA receptor agonists (MD-4.00, 95% CI [−12.84 to 4.84]), NMDA receptor antagonists (MD −4.22, 95% CI [−9.43 to .99]), and neurogenic and angiogenic agents (MD −6.00, 95% CI [−12.11 to .11]). The average number of days before discharge from the hospital was lower in each of these subgroups, and heterogeneity between the studies was not statistically significant (I^2^ = 0).

### 3.7 Risk of bias among included studies

**FIGURE 5:**
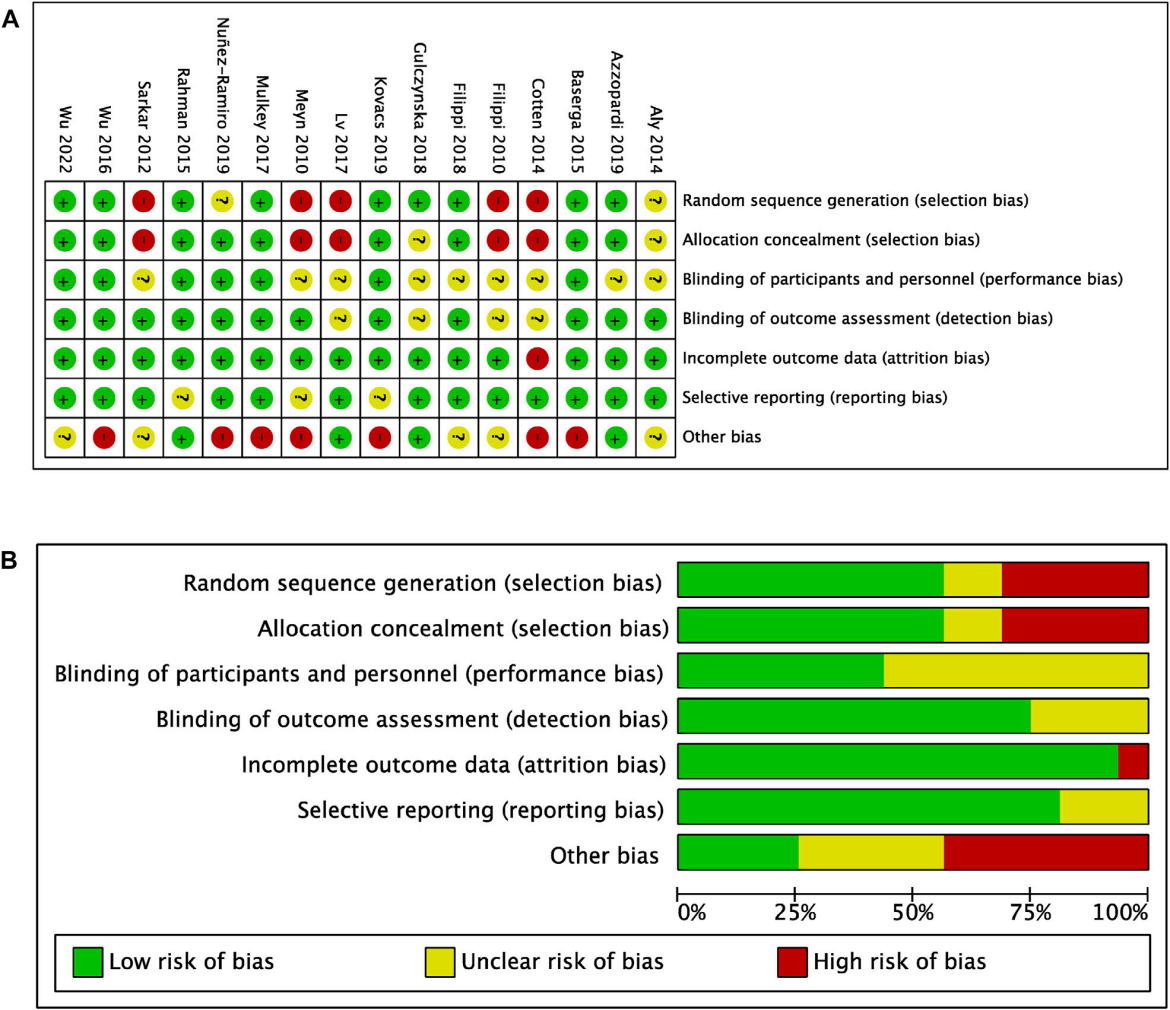
Risk of bias **(A)** Risk of bias summary **(B)** Risk of bias graph with authors’ judgements about each risk of bias item presented as percentages across all included studies.

The risk of bias assessment of all included studies is presented in Figure 5. Overall, studies had a moderate risk of selection bias due to lack of randomization into intervention groups, unclear sequence generation process for randomization, or unclear method of concealment of participants to randomized groups (high risk: 5/16; unclear risk: 2/16). Only 7/16 studies utilized placebos in the TH alone groups, however the authors judged that this was unlikely to influence outcome performance (unclear risk: 9/16). Furthermore, there was a low risk of detection (unclear risk: 4/16), attrition (high risk: 1/16) and reporting bias (unclear risk: 3/16), but a high risk of other bias (high risk: 7/16; unclear risk: 5/16). This was attributed to unclear TH protocols, inconsistent drug doses, use of supportive therapies, uncontrolled HIE severity among infants in both intervention groups, and uncontrolled numbers of in-born and out-born infants between the intervention groups, potentially allowing for select infants to reach target hypothermic temperatures quicker.

### 3.8 Publication bias

**FIGURE 6:**
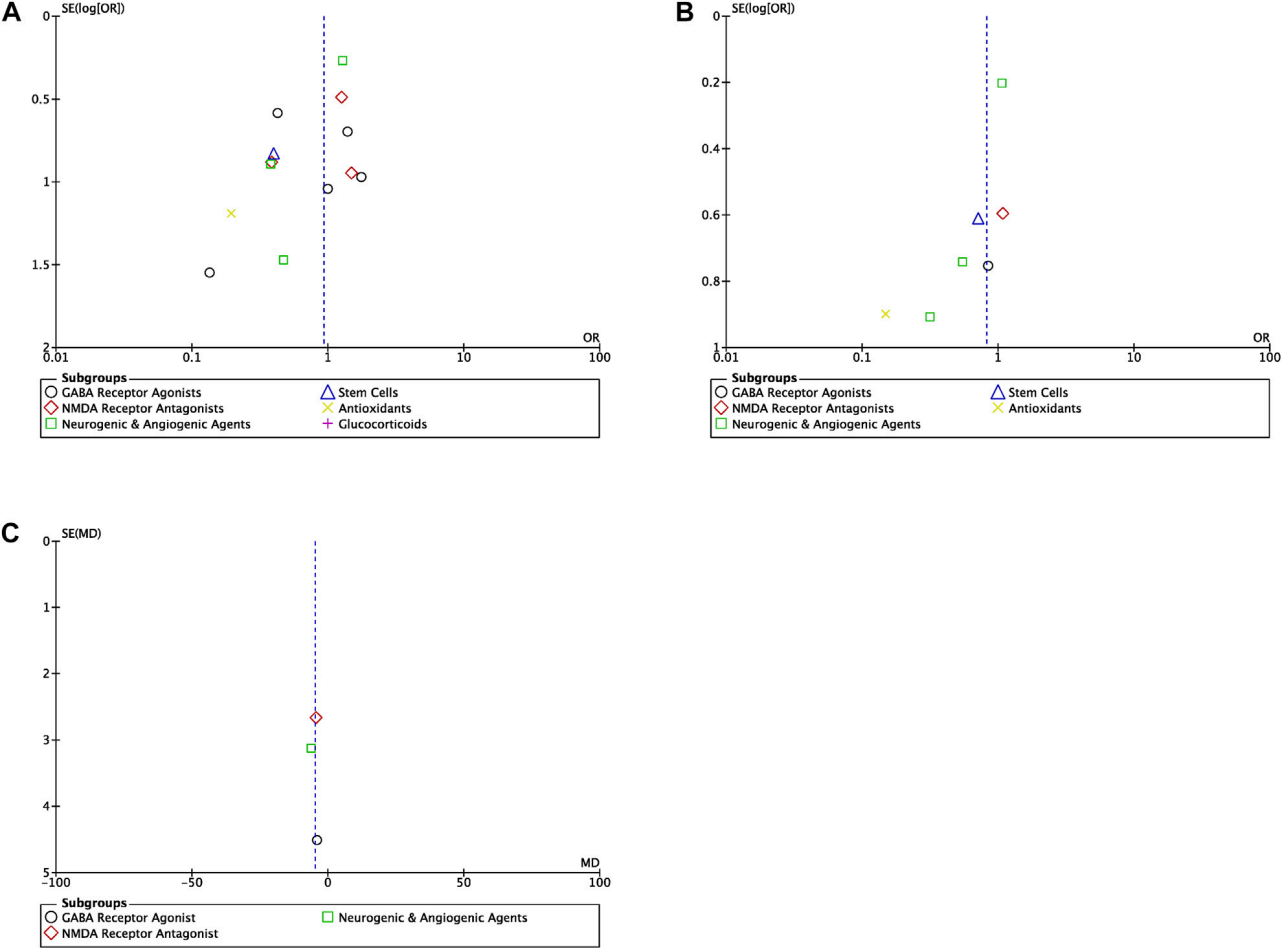
Funnel plots **(A)** Funnel plot for the studies reporting mortality for all drug classes (OR, odds ratio) **(B)** Funnel plot for the studies reporting NDI for all drug classes (OR, odds ratio) **(C)** Funnel plot for the studies reporting length of hospitalization for all drug classes (MD, mean difference).

Publication bias for each outcome–mortality, NDI and length of hospitalization–was not found to be a significant factor in the present meta-analysis (Figure 6). Symmetric scattering of the published data on either side of the overall effect size can be observed for each of the produced funnel plots.

## 4 Discussion

### 4.1 Summary of findings

Concerning the treatment of infants with moderate or severe HIE, the present analysis identified GABA receptor agonists, NMDA receptor antagonists, neurogenic and angiogenic agents, stem cells, glucocorticoids and antioxidants, as candidate adjuvants to TH that have been evaluated in clinical settings compared to TH alone. Meta-analysis revealed that HIE infants endure a significantly shorter duration of hospitalization post-birth when treated with TH and a neuroprotective adjuvant, compared to those treated with TH alone, which stands as the current standard of care (Table 3). Risk of mortality and NDI did not differ between combination therapy- and TH alone-treated infants. Due to a limited number of included studies, small sample sizes, and conflicting findings between studies, subgroup analysis did not reveal any particular class of drug to be a more efficacious adjuvant to TH than others.

**TABLE 3:** Summary of findings table for outcomes assessed. Effect sizes [odds ratio (OR): mortality, NDI; mean difference (MD): length of hospitalization], 95% confidence intervals (95% CI) and two-tailed *p*-values.

Population	Infants, gestational age ≥35 weeks, with evidence of moderate/severe HIE, both sexes, all ethnicity, all nationality		
**I**ntervention	Combined TH and therapeutic agent		
**C**omparison	TH alone		
**O**utcome	Mortality; NDI; length of hospitalization		
**S**tudy Design(s)	Randomized and quasi-randomized clinical trials, observational and retrospective studies		

Mortality: Combination Therapy vs. TH Alone			
Number of Studies	OR	95% CI	*p*-value
GABA Receptor Agonists			
5	0.76	[0.37, 1.57]	0.46
NMDA Receptor Antagonists			
3	1.03	[0.48, 2.22]	0.93
Neurogenic and Angiogenic Agents			
3	1.10	[0.62, 1.94]	0.75
Stem Cells			
1	0.40	[0.08, 2.01]	0.26
Glucocorticoids			
1	Not estimable		
Antioxidants			
1	0.02	[0.02,2.02]	0.17
Total			
14	0.93	[0.66, 1.32]	0.68

NDI: Combination Therapy vs. TH Alone			
Number of Studies	OR	95% CI	*p*-value
GABA Receptor Agonists			
1	0.85	[0.19, 3.69]	0.83
NMDA Receptor Antagonists			
1	1.1	[0.34, 3.51]	0.88
Neurogenic and Angiogenic Agents			
3	0.87	[0.50, 1.52]	0.64
Stem Cells			
1	0.72	[0.22, 2.39]	0.59
Antioxidants			
1	0.15	[0.03, .87]	0.03*
Total			
7	0.82	[0.55, 1.23]	0.34

Length of Hospitalization: Combination Therapy vs. TH Alone			
Number of studies	MD	95% CI	*p*-value
GABA Receptor Agonists			
1	−4.00	[−12.84, 4.84]	0.38
NMDA Receptor Antagonists			
1	−4.22	[−9.43, 0.99]	0.11
Neurogenic and Angiogenic Agents			
1	−6.00	[−12.11, 0.11]	0.05
Total			
3	−4.81	[−8.42, −1.19]	0.009**

OR, values below 1.00 denote decreased likelihood of death or NDI, to occur in combination therapy groups compared to TH, alone groups. Negative MD, values denote reduced days spent in hospital in combination therapy groups compared to TH, alone groups. *p < .05; **p < .01.

### 4.2 Proposed synergistic/additive neuroprotective mechanisms of adjuvants drugs and TH

**FIGURE 7:**
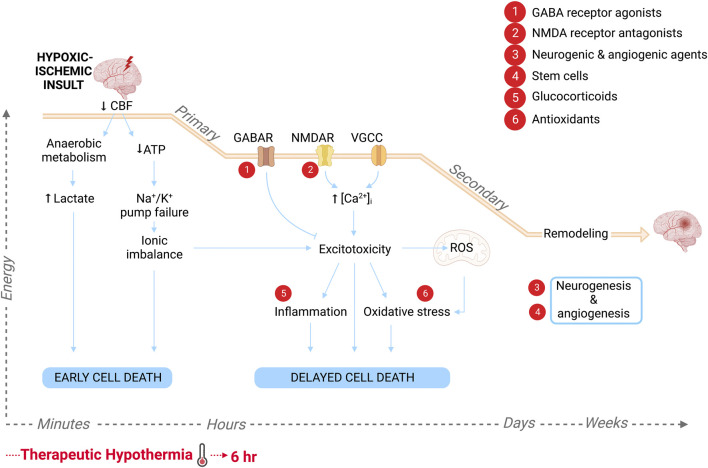
Schematic of the pathological progression in the brain following HI insult and the molecular targets of the various adjuvant therapies to hypothermia, labeled 1–6. Primary energy failure is characterized by a drop in cerebral blood flow (CBF) leading to lactate accumulation and high energy phosphate depletion. The resultant cytotoxicity triggers early cell death. Approximately 6–15 h later following a latent period due to reperfusion of brain tissue, secondary energy failure ensues. Neuronal depolarization leads to synaptic glutamate accumulation, causing excessive Ca^2+^ influx through NMDA receptors, voltage-gated Ca^2+^ channels (VGCC) and calcium permeable channels, as well as excessive release from intracellular stores. Excitotoxicity within the cell leads to the production of free radicals and mitochondrial dysfunction, causing oxidative stress, as well as inflammatory signaling. This ultimately leads to delayed neurodegeneration. Days to months after the initial insult, there is a tertiary phase characterized by repair, reorganization of neuronal circuits and new cell growth. Created with BioRender.com.

The outcome effect sizes and subgroup trends revealed through this meta-analysis nevertheless have important physiological and clinical implications for advancing the treatment of neonatal HIE. Although TH is a clinically proven safe and effective intervention modality, it alone is insufficient to protect against HIE-related mortality and morbidity hence the pursuit of a suitable drug to enhance its neuroprotective effects. Through this analysis we have identified drug targets under hypothermic temperatures that have been suggested to complement the molecular mechanisms underlying hypothermic-mediated protection. The benefit of TH comes from its multimodal targeting of injurious cascades during primary and secondary energy failure, that ultimately lead to irreversible neuronal death. Specifically, a reduction in physiologic temperature reduces cerebral metabolism, delaying the onset of anoxic depolarization and accumulation of excitotoxins (Bart et al., 1998; Wassink et al., 2014). In animal models of ischemia, hypothermia has also been shown to inhibit NO and superoxide formation, pro-inflammatory cytokine production and apoptotic cell death-mediators (Zhao et al., 2005; Zhang et al., 2010; Wassink et al., 2014). The mechanisms of the neuroprotectants used alongside of TH in this analysis overlap with this wide array of reported mechanisms of action of TH (Figure 7). In particular, GABA receptor agonists also suppress neuronal excitability while NMDA receptor antagonists reduce excitotoxicity, glucocorticoids inhibit neuroinflammation and antioxidants target oxidative stress. Thus, when used in combination with TH, we might expect synergistic inhibition against these specific pathological cascades. Moreover, while TH primarily targets the acute and secondary phases of HIE, as demonstrated by the strict therapeutic window, neurogenic and angiogenic agents as well as stem cell treatment, may enhance endogenous repair mechanisms during the recovery phase, leading to additive neuroprotective effects when used concurrently. These findings are important and can guide the direction of future preclinical studies that continue to delineate the complex molecular mechanisms that underlie HIE pathology.

### 4.3 Candidate adjuvants to TH: Optimal administration protocols within each drug class

Subgroup analysis revealed that combination therapies with GABA receptor agonists, stem cells and antioxidants, consistently trended towards more efficacious outcomes than TH alone. However, each subgroup had discrepancies, even between studies utilizing the same drug. This highlights the importance of optimizing administration protocols, particularly drug dose and duration. Within GABA receptor agonists, the two studies that combined PB with TH followed the same time course of administration but at different drug doses. PB at 40 mg/kg favored combination therapy (Meyn et al., 2010) while ∼25 mg/kg favored TH alone (Sarkar et al., 2011). Previous reports have demonstrated that in severe birth asphyxiated infants, 40 mg/kg PB alone was safe and well-tolerated, reduced cerebral spinal fluid lipid peroxide levels, and was associated with normal neurological outcome at 3 years of age in 73.3% of treated infants compared to 18.7% of untreated infants (Hall et al., 1998; Gathwala et al., 2011). However, PB has also been associated with neuronal degeneration in the immature brain, with 40 mg/kg representing the threshold dose for triggering apoptotic death (Bittigau et al., 2002). We speculate that in combination paradigms, hypothermia acted to suppress high-dose PB-induced apoptosis thereby nullifying potential adverse effects. However, the extent of hypothermic-mediated apoptotic suppression remains unknown. Thus, a larger single dose of PB may exert enhanced protection, but caution should be exercised if utilizing doses above 40 mg/kg, even in the presence of hypothermia. Moreover, three studies examined the effect of combined TPM and TH against infant mortality. Filippi et al. was the first trial to investigate the safety profile of TPM in HIE infants treated with TH (Filippi et al., 2010). TPM at low and moderate doses combined with either mild (32°C–34°C) or deep (30°C–33°C) TH was deemed safe, with no reported adverse biochemical or hemodynamic effects, and no differences in incidence of abdominal or ophthalmologic abnormalities as well as brain lesions compared with TH control groups. In terms of protocol and corresponding efficacy, each of the three studies included in this analysis administered TPM at the initiation of TH, however a loading dose of 5 mg/kg and maintenance dose of 3 mg/kg/dose for a total of 6 days, led to more favorable outcomes than the same loading and maintenance dose for a total of 3 days or 10 mg/kg/dose for 3 days (Filippi et al., 2010; 2018; Nuñez-Ramiro et al., 2019). Interestingly, at the beneficial dose and duration, 75.5% of infants reached serum therapeutic levels (TL) at 48 h of life, which was significantly correlated with a reduction in seizure activity. Although this would suggest that a higher loading dose would lead to earlier achievement of serum TL and thus more effective seizures control which often affects long-term outcome, higher doses such as 10 mg/kg or 25 kg/mg as reported by other studies, have failed to reduce mortality and morbidity (Glass et al., 2011). These findings thereby suggest that TPM administered at moderate doses for more days post-birth, confers more efficacious outcomes when combined with TH.

Regarding NMDA receptor antagonists, when combined with TH, MgSO4 at neither a 10% nor 20% concentration consistently reduced the likelihood of adverse outcome (Rahman et al., 2015; Gulczynska et al., 2018). These findings correspond with a previous meta-analysis of MgSO4 for infants with HIE which revealed increased mortality in MgSO4 treated-infants compared to controls (Tagin et al., 2013). Although antenatal administration of MgSO4 has a proven neuroprotective effect against cerebral palsy and gross motor dysfunction in preterm neonates, use in perinatal HIE animal models either with or without TH, delivers highly inconsistent results (Conde-Agudelo and Romero, 2009; Doyle et al., 2009; Galinsky et al., 2014). Thus, more rigorous testing at preclinical and clinical stages is needed before MgSO4 can be considered an effective monotherapy or adjuvant to TH for the treatment of HIE infants. Furthermore, although xenon gas has consistently showed promising neuroprotective effects when combined with TH in neonatal animal studies, few human trials have reported potent therapeutic effects, including the study included in this analysis (Ma et al., 2005; Hobbs et al., 2008; Azzopardi et al., 2019). An important caveat for the included study is that ventilation with xenon was only started within 6 h of birth in 15% of infants, while 11% began treatment after 12 h. This delay in administration is longer than what has been tested in animal models and thus may have been beyond the therapeutic window of xenon. Additionally, lack of a robust treatment protocol for humans and obstructed delivery of the agent through recirculating ventilated inhalation, highlights the necessity for increased clinical studies and protocol development for xenon gas before the relatively costly agent can be recommended as an adjunct to TH.

TH combined with neurogenic and angiogenic agents Epo, or its synthetic analog, Dpo, did not reduce the risk of mortality or NDI in HIE infants (Baserga et al., 2015; Wu et al., 2016; Lv et al., 2017; Wu et al., 2022). Combination therapy did however decrease the length of hospitalization (Mulkey et al., 2017). Pre-clinical and early small, pilot clinical studies reported that recurrent doses of Epo at 300–2500 U/kg had a safe pharmacokinetic profile and improved developmental outcome when administered in conjunction with TH (Zhu et al., 2009; Elmahdy et al., 2010; Wu et al., 2012). However these findings contrast the recent reports from the largest, and most robust RCT included in this analysis, which found that combined Epo and TH had no effect on the incidence of death or neurodevelopmental disability at 2–3 years of age, and furthermore increased the likelihood of having at least one serious adverse event compared to those treated with TH and a placebo (Wu et al., 2022). The safety concerns raised by this trial warrant further investigation into the optimal dosage and timing of Epo treatment under hypothermic temperatures. As Epo promotes recovery and repair, perhaps delayed administration following secondary energy failure, may exert additive protection to TH while minimizing potential toxicity. An ongoing large, RCT evaluating TH and Epo at 1000 U/kg may further inform our understanding of this potential adjuvant (PAEAN, Clinical Trials.gov, 2022a, NCT03079167). In a similar regard, the dose at which optimal neuroprotective effects are observed with Dpo treatment remains unknown; weekly Dpo administration at both low and high doses (2–10 ug/kg/dose) has been shown to produce sufficient serum Epo concentrations and favourable outcomes when utilized with TH (Baserga et al., 2015). A controlled comparison of Epo and Dpo is required.

Moreover, the regenerative properties of UCB cells–a potent source of stem cells and haematopoietic precursor cells–has attracted recent attention and are advantageous in that collection is non-invasive, poses no risk to the mother or infant and presents a low risk of infection transmission (Tsuji et al., 2020; Serrenho et al., 2021; del Pozo et al., 2022). There are currently nine clinical trials registered to evaluate intravenous infusion of autologous UCB-derived stem cells in neonatal HIE (Clinical Trials.gov, 2022b, NCT01649648, NCT00593242, NCT02612155, NCT02455830, NCT02256618, NCT02881970, NCT02551003, NCT03352310, NCT02434965) only two are published, one of which combined treatment with TH and is included in the present analysis (Cotten et al., 2014; Tsuji et al., 2020). Both published independent pilot studies followed comparable protocols for collection and preparation of the non-cryopreserved, RBC- and volume-reduced mononuclear fraction of cord blood cells, with doses ranging from 10^7^–10^8^, demonstrating safety and feasibility. However, risk of mortality and NDI in HIE infants treated with combined UCB cells and TH did not differ from those treated with TH alone (Cotten et al., 2014). It is our hope that the forthcoming publication of clinical data will elucidate the therapeutic potential of UCB cells as well as whether the efficacy of cell therapy and TH improve when administered concurrently.

Finally, an estimated effect size could not be produced for the glucocorticoid hydrocortisone, due to an equivalent incidence of death in both the combination therapy group and TH alone group (Kovacs et al., 2019). It is important to note however that this study did demonstrate the effectiveness of combined hydrocortisone and TH in increasing blood pressure in volume resistant hypotensive HIE infants compared to those treated with TH alone. HI insult and reperfusion have been associated with reduced myocardial perfusion and performance and thus the typically employed vasopressor-inotropes may be ineffective due to compromised cardiac output and further, actually stimulate negative compensatory mechanisms such as tachycardia (Zanelli et al., 2010; Giesinger et al., 2017; Diederen et al., 2018). Treatment with TH, which raises systemic vascular resistance, combined with hydrocortisone, led to reduced heart rate and duration of cardiovascular support and inotrope usage (Kovacs et al., 2019). This suggests hydrocortisone is an effective adjuvant to TH in the context of treating HIE-related hypotension. Lastly, combined TH and antioxidant, melatonin, significantly reduced the risk of NDI (Aly et al., 2014). Caution must be exercised when interpreting these results however as only one clinical trial was analyzed with a relatively small sample size. NDI was also reported at 6 months of age which may not be reflective of long-term outcomes. Human HIE studies evaluating melatonin as a monotherapy range in their timing of administration and dose (10 mg/kg—80 mg/kg), thus the optimal protocol parameters required to reach therapeutic levels remains unknown (Fulia et al., 2001; Ahmad et al., 2018). Although supportive pre-clinical animal data for the neuroprotective effects of melatonin in HIE is extensive, larger powered, efficacy RCTs of TH with melatonin at therapeutic levels and long-term follow up, are needed.

### 4.4 Strengths and limitations

The strength of this study lies in that it is, to the best of our knowledge, the first to evaluate the efficacy of combination therapy vs*.* TH alone in treating neonatal HIE. Identifying potential adjuvants to TH is a major focus of recent literature yet no study has measured whether such confers augmented neuroprotection at the clinical level in a broad sense. We have also categorized promising combination therapies by mechanism of action which highlights key targets in HIE pathology under hypothermic temperatures. Additionally, the present study analyzed three outcomes, mortality, NDI and length of hospitalization, which are important measures of the personal burdens posed by HIE as well as socioeconomic burdens placed on healthcare systems. Finally the included studies consist of infant data from a variety of research and clinical settings across the globe including Hungary (2.5%), China (3.2%), Poland (5.8%), the Middle East (7%), the United Kingdom (7.1%), Italy (7.6%), Spain (8.2%), and United States (58.5%). This diversity is representative of the translatability of our findings, particularly in high-income countries (HICs). Furthermore, there are two approved methods of TH induction utilized in HICs–selective-head and whole-body cooling. Selective-head cooling is achieved using a manually controlled cool cap fitted around the infant’s head, and whole-body cooling may be achieved *via* passive, environmental cooling, ice packs and/or commercially available cooling blankets. Outcomes at 12 months of age do not differ between HIE infants treated with either of the two methods, and both have been demonstrated to reduce the risk of death or major developmental disability compared to normothermia (Tagin et al., 2012; Celik et al., 2016). Comparable protocols used in each of the included studies in this analysis eliminates confounding bias.

The limitations of the present analysis arise predominantly from a limited number of available studies, and heterogeneity in the included studies’ design, intervention protocols, and assessed outcome measures. Seven of the studies were limited by small sample sizes of ≤50 infants (Meyn et al., 2010; Aly et al., 2014; Baserga et al., 2015; Wu et al., 2016; Lv et al., 2017; Filippi et al., 2018; Kovacs et al., 2019), while the large RCT conducted by Wu et al. accounted for 38.8% of all infants in the included analysis (Wu et al., 2022). There was no common assessment of HIE across studies and HIE severity information was missing entirely from four studies. Control for severity of encephalopathy at baseline between the treatment and control groups is essential, as it is correlated with infant death, disability and hospitalization, and is predictive of response to treatment. Additionally, timepoint of assessment for NDI varied from 6 months—3 years of age and the tests utilized to support an NDI diagnosis also differed between the studies. Although standardized assessments that measured motor, personal-social, language and adaptability domains were used, future analyses should evaluate the effect of combination therapy on specific neurological modalities to identify whether certain adjuvants reduce adverse outcomes in certain neurodevelopmental domains. Further, supportive agents were utilized alongside of the primary interventions as necessary, to provide respiratory assistance, hemodynamic support and seizure control. We therefore cannot conclude that any effects observed were solely attributed to either TH or the adjuvant therapeutic.

Finally, the outcomes of the present analysis are specific for HICs and are unlikely to be applicable for low-income and middle-income countries (LMICs). Although recommended by the International Liaison Committee Resuscitation guidelines in 2015 as the standard of care for neonatal encephalopathy in LMICs, the recent ‘hypothermia for neonatal encephalopathy in LMICs’ (HELIX) trial, reported TH to be ineffective (Perlman et al., 2015; Thayyil et al., 2021). The multi-country, rigorous RCT found that TH did not reduce the composite outcome of death or disability at 18 months of age and increased the incidence of death alone relative to a control group (Thayyil et al., 2021). In the HELIX trial, intra- and postpartum care were not standardized as 67% of included infants were born outside of the participating hospitals, with 2%–3% born at home. As well, at significantly higher rates than in HICs, 73%–74% of each cohort presented with clinical seizures at randomization and 80% had white matter damage, indicative of subacute injury (Miller et al., 2005). The quality of intrapartum and neonatal care as well as the subacute nature of neonatal brain injury in LMICs are important considerations in addressing the HIE burden in such settings and may partially underlie the lack of neuroprotection afforded by TH. Understanding the mechanisms by which the adjuvant drugs included in this analysis confer protection at the clinical level may nevertheless reveal novel therapeutics that are relevant in LMICs and warrant further investigation.

## 5 Conclusion

Moderate and severe HIE infants endure a significantly shorter duration of hospitalization post-birth when treated with TH and a neuroprotective adjuvant, compared to those treated with TH alone. GABA receptor agonists, NMDA receptor antagonists, neurogenic and angiogenic agents, stem cells, glucocorticoids and antioxidants represent promising candidate adjuvants that either target overlapping or additive pathophysiological mechanisms to TH. Despite compelling preclinical evidence however, risk of mortality and NDI did not differ between HIE infants treated with combination therapy and those treated with TH alone. This suggests a knowledge gap in clinically important therapeutic targets and how these candidate drugs interact with hypothermic-temperatures in clinical settings. It furthermore necessitates investigating the optimal dose and timing of administration at which maximal clinical benefits are observed for each adjuvant neuroprotectant and whether the efficacy of the neuroprotectant and TH are indeed enhanced when used in combination. The studies included in this analysis are limited in number and sample size, and are restricted to HICs. As TH is now the standard of care for HIE, it is important for preclinical trials to be conducting drug development studies in hypothermic settings and in turn, well-designed, larger powered trials from both HICs and LMICs are needed at the clinical level in order to streamline the translational pipeline and take combination therapy from bench to bedside.
